# Intercalative DNA binding of the marine anticancer drug variolin B

**DOI:** 10.1038/srep39680

**Published:** 2017-01-04

**Authors:** Albert Canals, Raquel Arribas-Bosacoma, Fernando Albericio, Mercedes Álvarez, Joan Aymamí, Miquel Coll

**Affiliations:** 1 Institute for Research in Biomedicine (IRB Barcelona), The Barcelona Institute of Science and Technology, Baldiri Reixac 10, 08028 Barcelona, Spain; 2 Institut de Biologia Molecular de Barcelona (CSIC), Barcelona Science Park, Baldiri Reixac 10, 08028 Barcelona, Spain; 3 CIBER-BBN, Networking Centre on Bioengineering Biomaterials and Nanomedicine, Barcelona Science Park, Baldiri Reixac 10, 08028 Barcelona, Spain; 4 Department of Organic Chemistry, University of Barcelona, 08028 Barcelona, Spain; 5 School of Chemistry, University of KwaZulu-Natal, 4001 Durban, South Africa; 6 Laboratory of Organic Chemistry, Faculty of Pharmacy, University of Barcelona, 08028 Barcelona, Spain; 7 Departament d’Enginyeria Química, Universitat Politècnica de Catalunya, Diagonal 647, 08028 Barcelona, Spain

## Abstract

Variolin B is a rare marine alkaloid that showed promising anti-cancer activity soon after its isolation. It acts as a cyclin-dependent kinase inhibitor, although the precise mechanism through which it exerts the cytotoxic effects is still unknown. The crystal structure of a variolin B bound to a DNA forming a pseudo-Holliday junction shows that this compound can also contribute, through intercalative binding, to either the formation or stabilization of multi-stranded DNA forms.

Variolin B (9-amino-5-(2-aminopyrimidin-4-yl)-1H-pyrido[3,4]pyrrolo[3,5-c]pyrimidin-4-one) is a natural anti-tumour and antiviral compound. Isolated originally from the Antarctic sponge *Kirckpatrickia variolosa*, this structure has a pyrido[3′,2′:4,5]pyrrolo[1,2-c]pyrimidine ring core bound to a 2-aminopyrimidine ring (Fig. 1a)^1^. It exerts pro-apoptotic activity, causes cell-cycle perturbations on several human cancer cell lines, and is active against Herpes simplex type I virus^2^,^3^. Variolin B and some derivatives, such as the more soluble deoxyvariolin B analogue (Fig. 1a), can now also be produced through various synthetic approaches^4^,^5^,^6^,^7^,^8^,^9^. The planar structure of the central aromatic ring system of variolin B is reminiscent of the tetracyclic DNA intercalator ellipticine^10^, another natural nitrogen-rich anti-cancer product. This observation initially led researchers to believe that the aforementioned cytotoxic effects of variolin B were produced by intercalative binding to DNA^2^. However, experimental evidence of variolin B acting as a cyclin-dependent kinase inhibitor (both on CDK1 and CDK2) moved the attention to its interaction with these essential proteins^3^,^11^. The crystal structure of variolin B in complex with CDK2/cyclin A supported these results and, consequently, variolin-based drug design progressed to new types of protein-targeted compounds such as meriolins^12^,^13^. These findings, along with the lack of conclusive DNA binding assays because of the insolubility of the compound, set aside the idea of variolin B being a DNA intercalator. Nevertheless, here we report the high-resolution structure of a complex showing that variolin B also can bind to a multi-stranded DNA in an intercalative fashion.

## Results and Discussion

**Figure 1:**
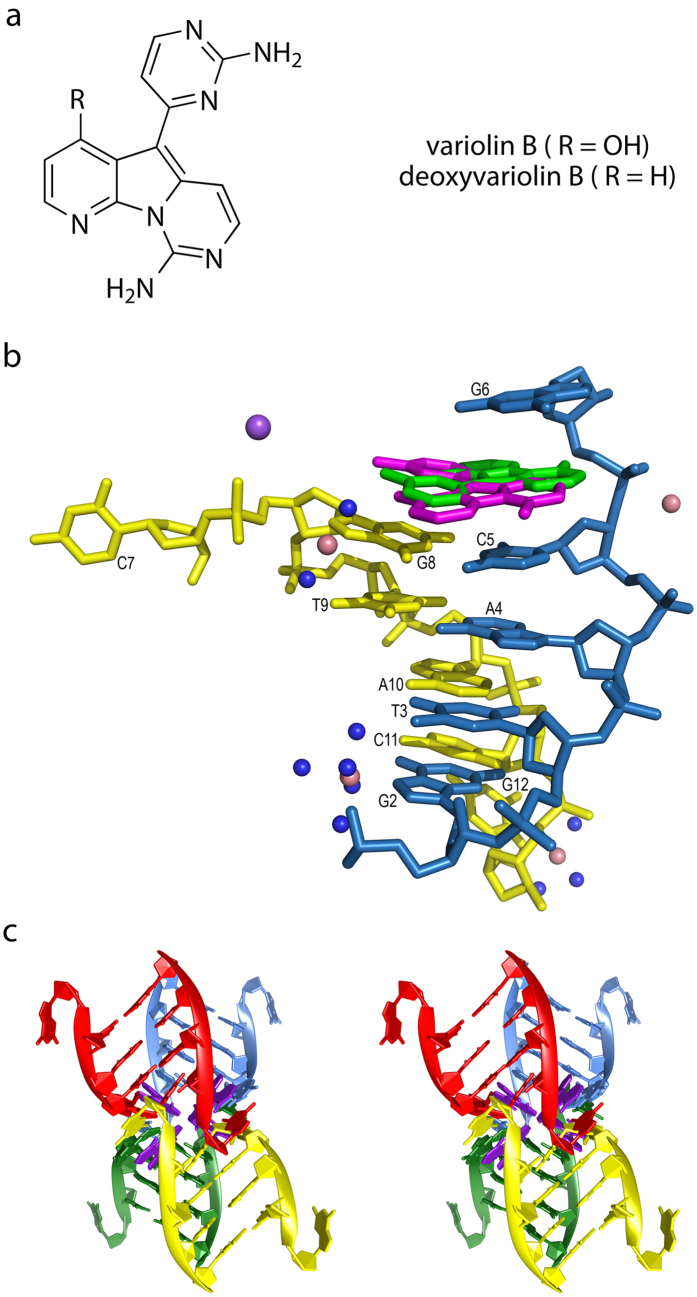
(**a**) Chemical structure of variolin B and the deoxyvariolin B derivative. (**b**) Content of the asymmetric unit of the d(CGTACG)_2_-variolin B crystal. Both half-occupancy drug molecules are shown. Cobalt and sodium atoms are represented with pink and purple spheres, respectively. Water molecules are not shown for clarity. (**c**) Stereoview of the four interlaced DNA duplexes (red, blue, green and yellow) forming four intercalation sites. One variolin B molecule is shown in purple in each of these sites.

**Figure 2:**
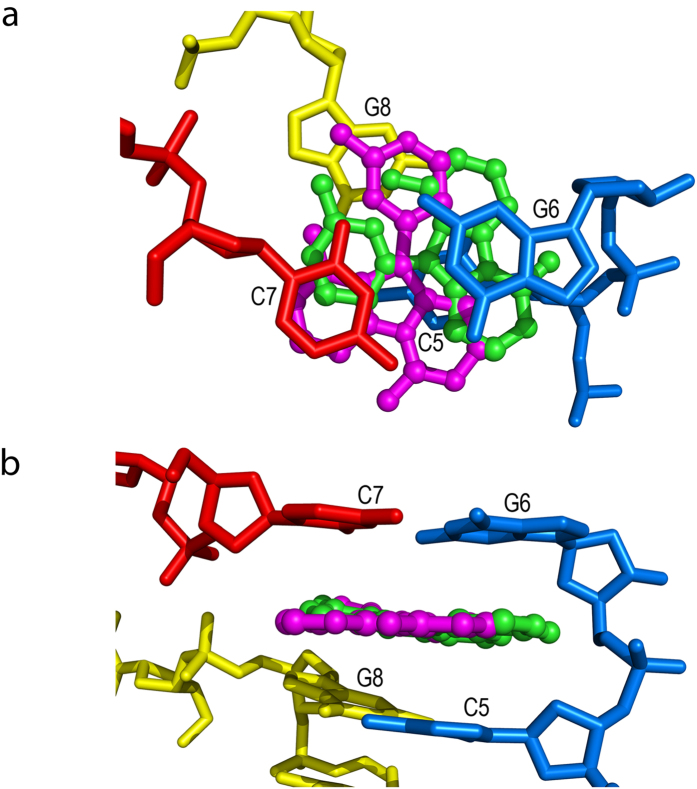
(**a**) Top and (**b**) side views of the intercalation site formed by the interduplex G6 · C7 and the intraduplex C5 · G8 base pairs, with the two sandwiched half-occupation variolin B molecules (green and magenta).

In the final model for the DNA-variolin B complex the DNA adopts a B-form conformation only along the four central base pairs of the d(CGTACG)_2_ duplex (Fig. 1b). As in previously reported isomorphous structures including Co^2+^ ions^14^,^15^,^16^,^17^,^18^, the strands at both ends of the DNA duplex are distorted and interact with symmetry-related DNA molecules. At one end cytosine C1 is disordered and the complementary guanine G12 is lying in the minor groove of a neighboring duplex. At the other end, cytosine C7 is rotated so that it pairs the guanine G6 of a symmetry-related duplex, forming a pseudo-Holliday junction^19^ that involves four DNA duplexes (Fig. 1c). Such association generates an intercalation site between the interduplex G6 · C7 and the intraduplex C5 · G8 base pairs (Fig. 2). While this particular crystal packing is caused mainly by DNA-DNA and DNA-ion interactions, a DNA intercalator is the keystone required to fix the whole structure.

The electron density for the intercalator is strong but does not allow to define a single orientation of the compound. Instead, the final model shows two drug molecules in different orientations, each with half occupancy. One of the orientations enables a bifurcated hydrogen bond between the −NH_2_ group of the tricyclic moiety of the drug and the guanine G6 phosphate.

Once intercalated, variolin B adopts a quasi-planar conformation (Fig. 2). The 2-aminopyrimidine ring is only 10.5° out of the pyridopyrrolopyrimidine mean plane in one drug molecule, whereas in the other this angle is 8.4°. Both conformations are consequently flatter than the one shown in the crystallographic structure of this small molecule alone^2^, where the external ring is 23.8° out of the heterocyclic core plane. Interestingly, in the structure of the CDK2/Cyclin A/variolin B complex^13^, the 2-aminopyrimidine ring of the drug is almost in plane with the heterocyclic system but flipped 180°. The interaction with CDK2, which probably determines this torsion, is based on three direct hydrogen bonds between variolin B and the ATP-binding site of the protein (residues Glu81, Leu83 and Ile10) and on several water-mediated hydrogen bonds.

This adaptability to several targets, either proteins or DNA, is rare but not unprecedented among similar natural alkaloids. Ellipticine, a proven DNA intercalator and topoisomerase II poison that is used in ovarian and breast cancer treatment, was also found to bind and inhibit protein kinase CK2 through competition with ATP^20^. In spite of this coincidence, the variolin B mode of binding to DNA cannot be paralleled to that of ellipticine^10^, as had been proposed^2^. Deoxyvariolin B affects the topoisomerase I-mediated unwinding of supercoiled DNA, but neither deoxyvariolin B nor variolin B increase the number of DNA strand breaks in a Comet assay with cells^3^. Hence, neither of these compounds seem to exert their cytotoxicity through DNA intercalation-mediated poisoning of topoisomerase I or II.

Variolin B is too insoluble to be reliably tested for its ability to bind DNA using absorption spectroscopy or surface plasmon resonance. However, its closely-related analogue deoxyvariolin B showed positive, albeit modest, binding to DNA using these techniques^3^. Variolin B also behaved as a weak DNA binder during our crystallization trials. After testing several oligonucleotides typically used to crystallize DNA-intercalator complexes, we observed variolin B only when binding to DNA folded into the intermolecular secondary structure described above. Taken together, these results suggest that variolins bind to DNA only under certain conditions, possibly involving the formation of non-B-form secondary structures. Variolin B intercalation stabilizes the unwinding of the DNA helix and the separation of the bases of the CpG dinucleotides, and also promotes the formation of the interduplex G6 · C7 base pair. The insertion of the drug is aided by the formation of a hydrogen bond between one of the NH2 groups of variolin B and the G6 phosphate. As a result of the rearrangement, the N7 atoms of guanines G6 and G8 are no longer able to interact with Co^2+^ ions^17^, which would probably impair the formation of the observed multi-stranded DNA structure.

As we have shown, variolin B is fully capable of disturbing B-DNA, promoting the formation of stable inter-helix junctions, just as other potent intercalators do^14^. This finding supports the notion that variolin B is a dual-action drug and suggests that its ability to stabilize nucleic acid junctions (either of DNA or RNA) might be further exploited^21^,^22^.

## Conclusions

Our structure confirms that variolin B is indeed a DNA intercalator. Therefore, a dual action of variolin B on both proteins and nucleic acids is most likely to occur. The cytotoxic effects of variolin B would then arise from a combination of its capacity to interact with crucial ATP-binding proteins and its ability to promote the formation of high-order nucleic acid systems in the cell environment.

## Methods

### Crystallization and data collection

Variolin B was synthesized as published elsewhere^4^. The d(CGTACG)_2_ synthetic DNA was purchased from Biomers.net. Crystallization conditions were screened using the Nucleic Acid Mini Screen^23^. Yellow bar-shaped crystals were grown at 293 K in a sitting-drop vapour-diffusion set-up by mixing 1.0 μl 5 mM variolin B in 25% v/v DMSO, 1.0 μl 3 mM d(CGTACG)_2_ and 2.0 μl crystallization solution (8% v/v 2-methyl-2,4-pentanediol, 40 mM sodium cacodylate pH 5.8, 4 mM hexammine cobalt, 12 mM sodium chloride, 80 mM potassium chloride) equilibrated against 500 μl 70% MPD. A dataset was collected on a MarMosaic 225 CCD detector (MAR Research) from a single crystal kept at 120 K using synchrotron radiation (λ = 0.8726 Å) at the ESRF (Grenoble) microfocus beamline ID 23-2. Data to 1.4 Å resolution were indexed, integrated, and scaled with the XDS package^24^ (Table 1).

### Structure solution and refinement

The structure was solved by molecular replacement with Phaser^25^ using the DNA coordinates from the structure of 9-amino-[N-(2-dimethylamino)propyl]-acridine-4-carboxamide bound to d(CGTACG)_2_ (PDB entry 1RQY)^14^ as a search model. Refinement followed with REFMAC5^26^. The optimum orientations of the intercalated variolin B were identified by placing the drug in each of the four possible positions and refining until the best fit and corresponding best R-factor and R-free were found. At this stage, an iterative refinement procedure was carried out using REFMAC5, interspersed with inspection of electron-density maps, water positioning, and manual model rebuilding with COOT^27^. All data were used (40.0–1.4 Å) with no resolution or σ cut-off. The final refinement statistics are shown in Table 1.

## Additional Information

**How to cite this article**: Canals, A. *et al*. Intercalative DNA binding of the marine anticancer drug variolin B. *Sci. Rep.* **7**, 39680; doi: 10.1038/srep39680 (2017).

**Publisher's note:** Springer Nature remains neutral with regard to jurisdictional claims in published maps and institutional affiliations.

## Figures and Tables

**Table 1:** Data collection and refinement statistics.

Data collection	
Space Group	C222
Unit-cell dimensions (Å)	*a* = 28.7, *b* = 53.4, *c* = 40.8
Wavelength (Å)	0.8726
Resolution Range (Å)	40.8–1.4
Rmerge (%)a,^b^	9.5 (19.0)
Mean I/σ(I)^a^	10.1 (5.0)
No. unique reflections	6465
Completeness (%)^a^	99.9 (100)
Redundancy^a^	18.2 (19.5)
**Refinement**	
Resolution (Å)	40.8–1.4
R-factor (%)^c^	20.8
R-free (%)^c^	25.3
No. atoms	309
Average B (Å^2^)	19.54
Asymmetric unit contents	1 DNA duplex
	1 drug (variolin B)
	3 Cobalt hexamine (III)
	1 Co^2+^
	1 Na^+^
	25 H_2_O

^a^Values for the highest resolution shell (1.8–1.4 Å) are in parentheses.

^b^Rmerge = ∑_*hkl*_∑_*i*_ |*I*_*i*_(*hkl*) − 〈*I(hkl*)〉|/∑_*hkl*_∑*I*_*i*_(*hkl*), where *I*_*i*_(*hkl*) is the *i*th observed amplitude of reflection *hkl,* and *I(hkl*) is the mean amplitude for all observations *i* of reflection *hkl*.

^c^R-factor = R-free = ∑_*hkl*_ ||*F*_obs_| − *k*|*F*_*calc*_||/∑*hkl*|*F*_obs_| calculated for the reflections of the working and test (5%) sets, respectively.
